# Exiguous premeal saccharide intake reduces subsequent food intake in men

**DOI:** 10.1007/s00394-021-02563-7

**Published:** 2021-04-23

**Authors:** Juliane Richter, Narona Thordsen, Kai Duysen, Kerstin M. Oltmanns

**Affiliations:** Section of Psychoneurobiology, Center of Brain, Behavior and Metabolism (CBBM), University of Luebeck, Ratzeburger Allee 160, 23562 Luebeck, Germany

**Keywords:** Satiety, Carbohydrate, Preload, Energy consumption, Blood glucose, Insulin

## Abstract

**Purpose:**

Satiety is a crucial factor in the attempt to reduce food intake for long-term body weight loss. Since there is evidence for a negative correlation between cerebral energy levels and food intake, the provision of the primary energy substrate glucose to the brain through oral ingestion of carbohydrates could trigger feelings of satiety. Therefore, we hypothesized that a low-calorie saccharide preload would increase satiety, reduce subsequent food intake, and thereby decrease overall calorie consumption.

**Methods:**

In a randomized single-blind crossover study, 17 healthy young normal-weight men received saccharide (26 kcal in total) or placebo capsules 30 min before a standardized breakfast buffet. We analysed food intake from the test buffet as well as plasma glucose and serum insulin levels.

**Results:**

The saccharide preload reduced food intake from the buffet by 168 (± 34) kcal (*p* < 0.001) compared to control. This corresponds to a net reduction in total calorie consumption by 142 (± 34) kcal (*p* < 0.001) or 9.3% due to saccharide capsules.

**Conclusion:**

A very low-calorie saccharide preload considerably reduces subsequent food intake leading to decreased overall calorie consumption. A saccharide preload before meals could, therefore, be a promising support for reducing caloric intake.

**German Clinical Trials Register:**

DRKS00010281 (date of registration: 11.04.2016)

## Introduction

Although reducing food intake is a self-evident strategy to combat overweight and obesity, the long-term implementation of changed behavior is difficult for many people as a caloric restriction often results in increased hunger and reduced satiety [1]. Satiety plays a pivotal role in the physiological regulation of food intake [2]. In complex interactions with peripheral mechanisms, central nervous system appetite regulation in the brain is of decisive importance [3] as food intake and energy balance are regulated inter alia through glucose-sensing neurons in the hypothalamic appetite centers. Since this mechanism is tightly linked to the current status of cerebral energy levels [4], it is conceivable that a carbohydrate preload before a meal could trigger feelings of satiety and thereby may reduce subsequent food intake.

In this context, previous studies examining the compensatory effect of a pure carbohydrate preload prior to food intake applied rather considerable amounts of glucose, fructose, sucrose, or maltodextrin with a calorie content between 100 and 360 kcal and a random time interval of 30–60 min between preload and test meals [5–8]. However, since blood glucose concentrations rapidly drop again after ingestion of such carbohydrates, any saturating effects on central nervous system appetite centers 1 h after application may be questioned. In this respect, a meta-analysis showed that the compensatory effect of a preload decreases with the increasing time interval between the preload and the test meal and with the increasing energy content of the preload [9].

We hypothesized that an exiguous amount of carbohydrates consisting of different saccharides causes a sustained but moderate increase in blood glucose levels preferably within fasting values, and thereby cerebral energy supply, and reduces subsequent food intake leading to a net calorie saving in the end. To ensure both rapid and sustained elevation of blood glucose levels, we composed a carbohydrate preload of mono and oligosaccharides and applied it in a placebo-controlled single-blind crossover design to healthy men to investigate the effects of a low-calorie saccharide composition as preload on food consumption 30 min after intake. As a distraction to divert participants from testing food intake from the buffet, an attention and concentration test was conducted before and after administration.

## Research design and methods

### Participants

We examined a group of 17 healthy young men (23.0 ± 0.7 years) with a normal body mass index (BMI 22.5 ± 0.4 kg/m^2^). Participants had a self-reported regular sleep–wake cycle. Exclusion criteria were acute or chronic internal, neurologic or psychiatric diseases, current medication of any kind, overweight (BMI > 26 kg/m^2^), alcohol or drug abuse, smoking, shift work and extraordinary mental or physical strain. Because the buffet was standardized for an omnivorous diet, men with special diets, such as vegetarians or vegans, were not included in the study. We also excluded competitive athletes as well as people with type 2 diabetes mellitus in first-degree family members. Volunteers did not participate in other studies or donate blood for 8 weeks before and during the experiments. The day before the experiments, participants were instructed not go to bed later than 11:00 PM and to fast at least 12 h prior to testing. Each participant gave written informed consent. The study has been approved by the ethics committee of the University of Luebeck and carried out in accordance with the Declaration of Helsinki of the World Medical Association. This trial was preregistered at German Clinical Trials Register (DRKS00010281).

### Experimental design and intervention

**Fig. 1:**
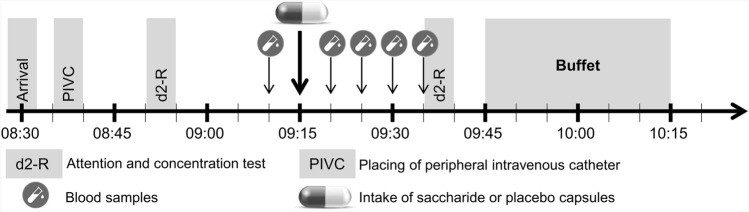
Schematic illustration of the experimental study design. Subjects participated in a saccharide and a placebo session separated by a minimum of 1 week

In a randomized single-blind crossover design, subjects participated in a saccharide and a placebo session separated by a minimum of 1 week (maximum: 5 weeks, mean: 2 weeks). The experimental design is illustrated in Fig. 1. The order of the two conditions was randomized using random numbers in Excel 2010. Participants were blinded in terms of the type of the capsule administered and in terms of food intake as the primary outcome. Investigators conducting the experiments were not blinded. One participant was tested at a time. On the days of experimental testing, the participant arrived at 8:30 AM at our human laboratory and the subject’s body weight was measured. After placing an intravenous forearm catheter, first blood samples were taken at 9:10 AM to determine baseline concentrations of glucose and insulin. Four further blood samples were taken (*t* = 5, 10, 15, 20 min after capsule ingestion).

Five minutes after baseline blood sampling, saccharide or placebo capsules were ingested with a glass of water (standardized 300 ml). At 9:45 AM a standardized breakfast buffet (composition shown in Table 1) was offered, from which participants were allowed to eat ad libitum during the subsequent 30 min. Based on experience from previous studies [10, 11], a time interval of 30 min is optimal for a meal from a test buffet. All participants completed their food intake within these 30 min. A longer time at the buffet could incite participants to boredom eating. Food intake was subsequently quantified and analyzed by weighing buffet components before and after food consumption. Participants were kept unaware of this procedure. In terms of the study goal, they were informed that the effects of saccharide ingestion on mental concentration and attention should be investigated. Therefore, attention and concentration tests (d2-R [12]) were performed 25 min before and after ingestion of the capsules, respectively. The test consists of 14 lines, in which the subject should strike out every letter “d” with two dashes within 20 s and ignore all other distractors. Three main characteristics were evaluated: (1) the number of correct responses minus commission errors, (2) the total number of symbols processed, and (3) the percentage of all (commission and omission) errors made within all symbols processed.

**Table 1:** Composition of the offered free-choice test buffet

Food	Weight (g)	Energy (kcal)	Carbohydrates (g)	Fat (g)	Protein (g)
Apple	150	98	22	0	0
Apple juice	200	88	21	0	0
Apricot jam	50	126	30	0	0
Bacon	45	68	0	3	9
Banana	170	158	34	0	2
Butter	75	556	0	62	1
Butter cheese	60	181	0	14	13
Cereals	60	206	37	2	6
Chocolate pudding	100	110	17	3	3
Cocoa drink	500	265	46	1	18
Coffee cream	40	49	2	4	1
Cream cheese	34	99	1	10	2
Cream cheese with herbs	40	105	1	10	3
Cream fruit yoghurt	150	246	28	12	5
Cucumber	70	10	1	0	0
Fruit quark	200	236	32	8	9
Honey	40	122	30	0	0
Marble cake	110	510	57	30	6
Margarine	70	496	0	56	0
Meatballs	100	260	10	19	13
Multigrain bread rolls	150	401	77	2	12
Natural yoghurt	150	57	8	0	6
Nut nougat cream	40	231	22	15	1
Orange juice	200	86	18	0	0
Poultry sausage	30	46	0	2	8
Salami sausage	22	88	0	8	4
Scramble eggs	100	154	1	11	13
Strawberry jam	50	130	31	0	0
Sugar	24	97	24	0	0
Tomato	100	20	3	0	1
Vanilla pudding	100	107	17	3	3
Wheat bread rolls	120	349	67	2	12
White bread	60	149	29	1	5
Whole-grain bread	120	314	38	8	11
Whole milk	500	320	24	18	17
Total	–	6538	728	304	184

The breakfast buffet was served with unsweetened tea and coffee

### Saccharide application

Saccharide composition consisted of honey powder and maltodextrin (NUPP nutrition, Oakwood & Son UG (limited liability), Lübeck, Germany). The saccharide mixture in the 14 capsules administered had a total energy content of 26 kcal and a weight of 6.8 g. Of the saccharide mixture administered, 3.3 g (48%) was honey powder consisting of 1.5 g of fructose, 1.2 g of glucose, 0.1 g of sucrose, 0.3 g of maltose, and 0.2 g of other ingredients (including fiber, amino acids, proteins, minerals, vitamins). The remaining 3.5 g (52%) of the mixture was maltodextrin (dextrose equivalent DE 10, degree of polymerization DP 10) made from corn starch. Placebo capsules contained cellulose, which was chosen as insoluble fiber to preclude effects on blood glucose levels. Saccharide and placebo capsules did not differ in administered number, weight, size, shape, color, and taste.

The appropriate number of saccharide capsules to affect brain energy homeostasis and the optimal time interval between capsule intake and test buffet was determined within the scope of a pilot study preceding the experiments. By means of ^31^Phosphorus magnetic resonance spectroscopy [11, 13], we measured increases in the cerebral high-energy phosphates adenosine triphosphate and phosphocreatine in 7.5-min intervals for the duration of 1 h after capsule intake. Two different dosages of saccharide intake were tested: 14 capsules (26 kcal) vs. 8 capsules (15 kcal). The aim was to increase blood glucose concentrations within fasting values but elevate high-energy phosphates, i.e., exert a neuroenergetic effect. Blood glucose concentrations were regularly measured to determine an increase due to saccharide application (B-Glucose-Data-Management, HemoCue GmbH). Results indicate a more sustained increase in cerebral high-energy phosphates and blood glucose levels upon 14 compared with 8 capsules. Peaks in all parameters were recorded within 25 and 35 min after capsule intake.

### Assays

Blood was centrifuged within 5 min after withdrawal and serum as well as plasma supernatants were kept at − 80 °C until assay. Serum insulin concentrations were measured by electrochemical luminescence immunoassay (ECLIA, Roche) with an intra-assay coefficient of variation (CV) < 5.3% and an inter-assay CV < 2.0%. Plasma glucose levels were measured by the hexokinase method (Beckman Coulter) with an intra-assay CV < 0.7% and an inter-assay CV < 1.3%.

### Statistical analysis

Sample size was calculated for a paired two-sided *t *test using the free software R (version 3.2.0). Based on previous test-buffet studies [14, 15], we assumed a value of 150 kcal with a standard deviation of 200 kcal for the smallest difference in food intake to be detected, and a power of 0.8 for an *α *error probability of 0.05. This calculation resulted in a minimum sample size of 16 subjects.

Data are presented as mean values ± standard error of mean (SEM) unless otherwise stated. Statistical analyses were based on analyzes of variance (ANOVA) for repeated measurements including the factors *treatment* (saccharide vs. placebo) and *time* (time points of data collection), as well as the *interaction* effect between these factors. In case of violation of the sphericity conjecture, Greenhouse–Geisser correction was applied. For pairwise comparisons, paired Student’s *t *test was used. *P* values less than 0.05 were considered significant. All calculations were done using SPSS Statistics 22 (IBM SPSS). In the analysis of serum insulin, one subject was excluded due to outlier values, i.e., increased insulin concentrations at all time points in the absence of elevated glucose concentrations. One participant was excluded from d2-R test analyses because testing was not correctly performed.

## Results

### Systemic glucose metabolism

**Fig. 2:**
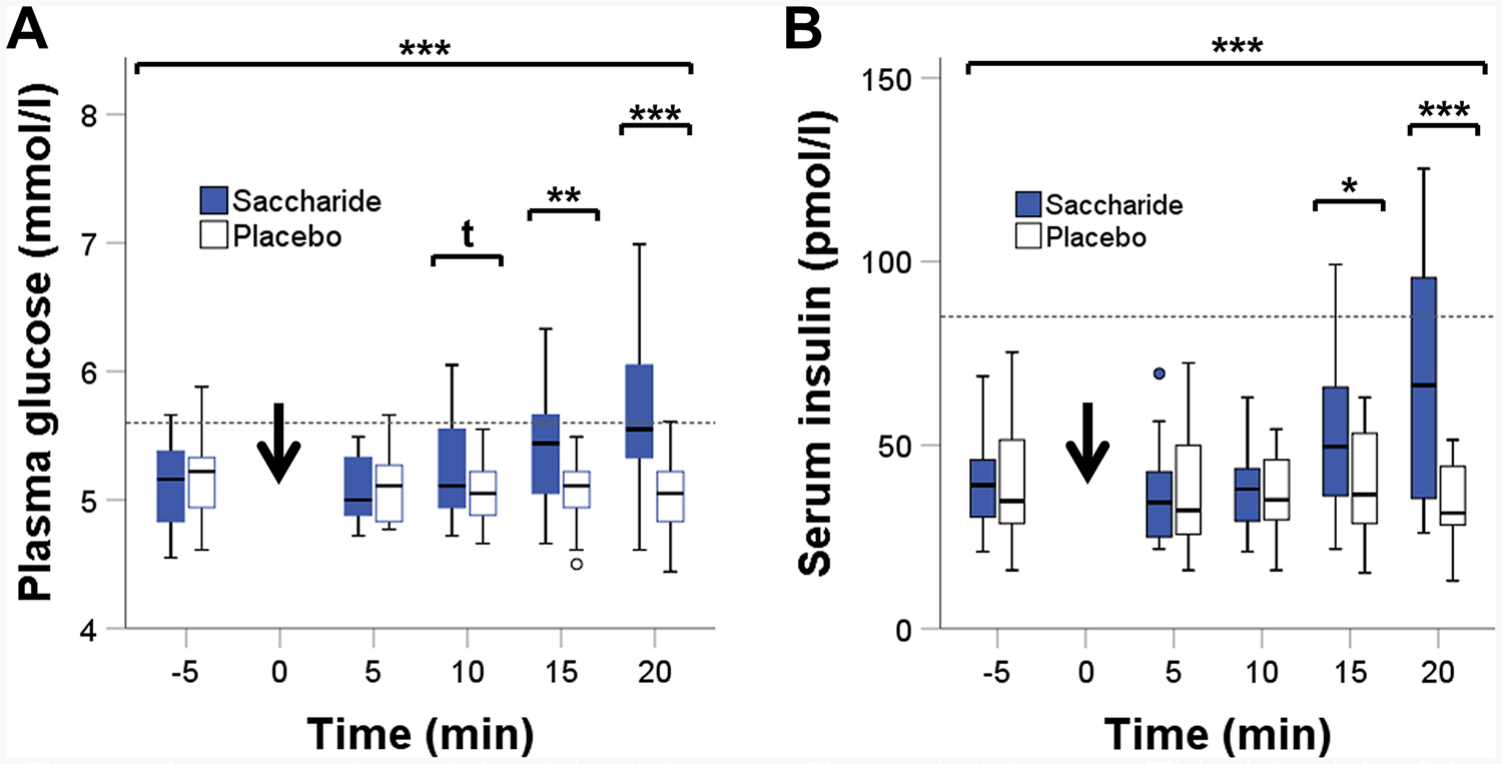
Concentrations of **a** plasma glucose (*n* = 17), and **b** serum insulin (*n* = 16) in the saccharide (blue boxplots) and the placebo (white boxplots) condition. The gray dashed lines reflect the upper norm values for fasting plasma glucose (**a**) and serum insulin (**b**) concentrations. The black arrow marks the intake of saccharide or placebo capsules. Line plots above the graphs show differences and interaction effects (*t*: *p* < 0, *p < 0.05, **p < 0.01, ***p < 0.001)

As expected, there were no differences in baseline plasma glucose and serum insulin levels between the two conditions (*p* > 0.517 for both, *t* tests, Fig. 2a, b). Similarly, within 10 min after ingestion of the capsules, insulin concentrations did not differ between conditions (*p* > 0.880, *t* test, Fig. 2b). In contrast, there was a non-significant trend for elevated blood glucose levels 10 min after the ingestion of the saccharides compared with placebo capsules (*p* = 0.052, *t *test, Fig. 2a). At 15 and 20 min after oral administration of the saccharide composition, both plasma glucose and serum insulin concentrations were significantly increased compared with placebo (*p* < 0.014 for all, *t* tests, Fig. 2a, b). This increase in glucose and insulin concentrations occurring only in the saccharide condition is reflected by significant interaction effects between treatment and time (*p* < 0.001 for both, *interaction* effects, Fig. 2a, b). Interestingly, despite these increases in plasma glucose and serum insulin concentrations within the first 20 min in the saccharide condition, plasma glucose concentrations were only slightly above the fasting reference value of 5.6 mmol/l and serum insulin concentrations remained within the range of normal fasting values (Fig. 2a, b).

### Food intake

**Fig. 3:**
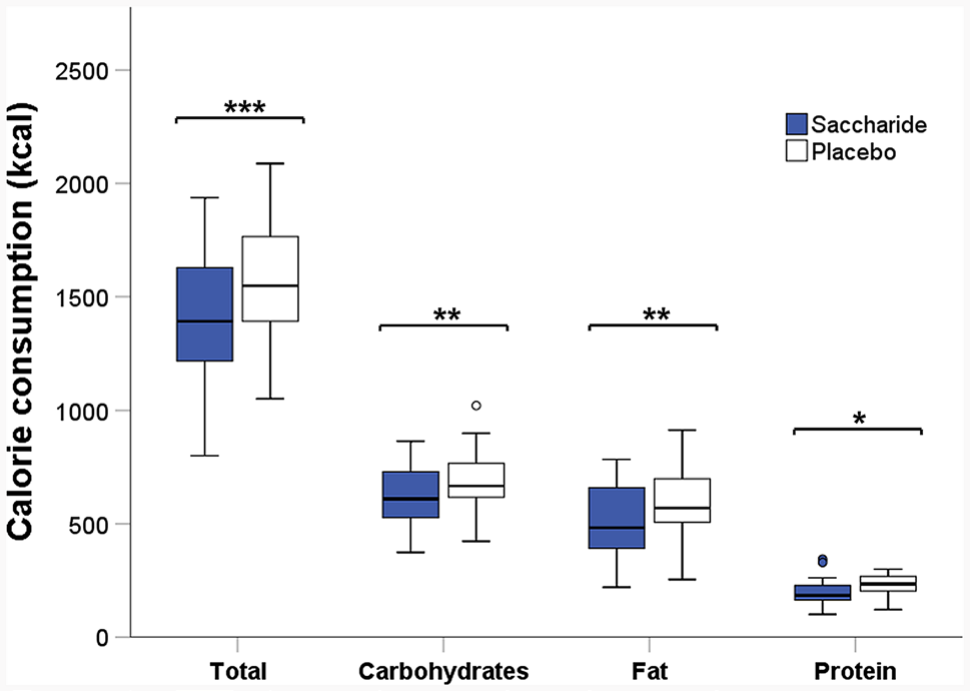
Calorie consumption in the saccharide (blue boxplots) and the placebo (white boxplots) condition. Line plots above the graphs show differences with statistical significance (*p < 0.05, **p < 0.01, ***p < 0.001, *n* = 17)

Effects of saccharide vs. placebo capsules on food intake behavior are shown in Fig. 3. In the saccharide condition, subjects consumed a total of 1350 ± 77 kcal, whereas in the placebo condition, total caloric intake was 1518 ± 70 kcal. This represents a significant reduction in total caloric intake of 11.1% by saccharide capsules (*p* < 0.001, *t* test, Fig. 3). Carbohydrates accounted for the largest portion of caloric food intake, with a significantly lower carbohydrate intake in the saccharide compared with the placebo condition (− 9.7%, *p* = 0.009, *t* test, Fig. 3). After intake of saccharide capsules, significantly less energy was consumed from fat than after placebo intake (− 12.3%, *p* = 0.002, *t* test, Fig. 3). In addition, there was a significant reduction in caloric intake from protein in the saccharide compared with the placebo condition (− 11.9%, *p* = 0.015, *t *test, Fig. 3).

Considering the energy content of the saccharide capsules in the analysis of food intake, there remains a highly significant difference of 142 ± 34 kcal in total calorie consumption between saccharide and placebo condition (− 9.3%, *p* < 0.001, *t *test including energy content of capsules). The difference in carbohydrate consumption was not significant after addition of the capsules’ calorie content (*p* = 0.088, *t* test). Since the saccharide preload contained neither protein nor fat, the consideration of the saccharide capsules’ energy content had no effect on the differences between conditions in terms of protein and fat consumption.

Energy density of the foods consumed was 1.6 ± 0.1 kcal/g in the saccharide condition and 1.5 ± 0.7 kcal/g in the placebo condition. There was no significant difference in the energy density between the two conditions (*p* = 0.707, *t* test).

### Attention and concentration testing (d2-R)

**Fig. 4:**
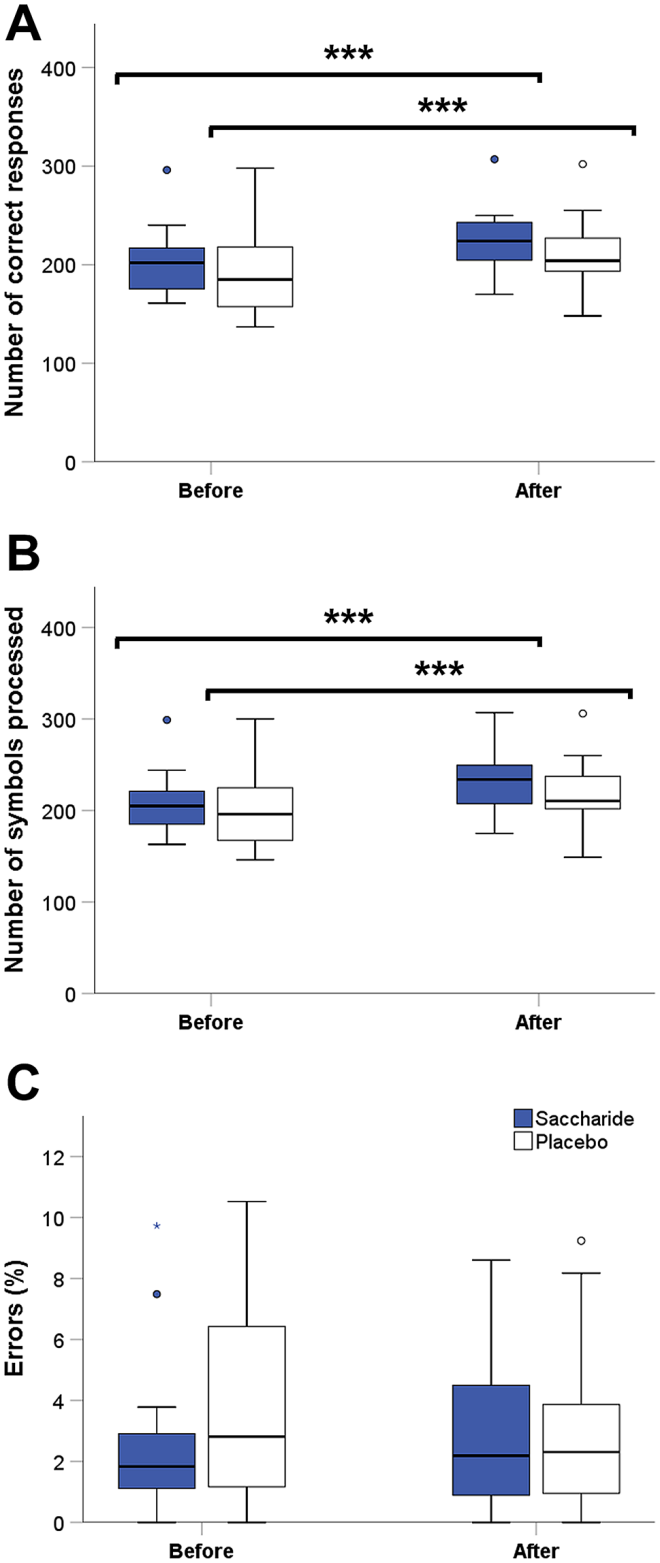
**a** Number of correct responses, **b** Number of symbols processed, and **c** percentage error of the d2-R test in the saccharide (blue boxplots) and the placebo (white boxplots) condition. Line plots above the graphs show time effects with statistical significance (***p < 0.001, *n* = 16)

There were no differences between saccharide and placebo condition at baseline in terms of the number of correct responses (*p* = 0.214, *t *test), the number of symbols processed (*p* = 0.283, *t *test), and the percentage of all errors (*p* = 0.177, *t *test, Fig. 4a–c). In both conditions, there were improvements in the number of correct responses (*p* < 0.001, *time* effects, Fig. 4a) and the number of symbols processed (*p* < 0.001, *time* effects, Fig. 4b) during the second testing 25 min after capsule ingestion compared with the first run. However, these improvements did not differ between the saccharide and the placebo condition (*p* = 0.970 for the number of correct responses, *p* = 0.684 for the number of symbols processed, *interaction* effects). In terms of the percentage of all errors there were neither improvements at the second test run compared with baseline (*p* > 0.344, *time* effects) nor any interactions between treatment and time (*p* = 0.213, *interaction* effect).

## Discussion

Our results show that a low-calorie saccharide preload preserving fasting values of blood glucose and insulin reduces subsequent food intake by 11.1% compared to placebo. After considering the calorie content of the saccharide application dosage of 26 kcal, the net reduction in overall calorie consumption is 9.3%, i.e., 142 kcal. Differential analyses revealed that the ingestion of saccharide capsules suppresses protein and fat consumption during the test buffet. This finding differs from previous studies, which predominantly found a higher total energy intake, i.e., preload plus test meal, after a considerable carbohydrate application prior to a meal [5–8, 16–18]. However, these divergent effects may result from fundamental differences in the study design in terms of the calorie amount and saccharide composition of the preload as well as the timing of capsule administration. Moreover, our approach followed a new hypothesis, which assumes that already an exiguous amount of glucose may increase blood glucose levels and thereby cerebral energy supply sufficiently to induce hypothalamic satiety perception and consequently reduce food intake. Indeed, our pilot study implied that even a small amount of 26 kcal is sufficient to raise blood glucose levels modestly and to increase the cerebral energy content. In contrast, previous studies testing the effects of a carbohydrate preload on food intake were mainly carried out under the hypothesis that the increase in sugar-sweetened beverage consumption is the cause of the raising prevalence of overweight and obesity [8]. Accordingly, in those studies, the calorie content of the preload was often based on the calorie content of a usual amount of customary soft drinks (1 glass, 1 can) and comprised considerable energy quantities between 100 [6] and 360 kcal [8]. Only one study found that a sucrose preload of 100 kcal resulted in a significantly lower total energy intake during a pizza test meal served 60 min after preload ingestion compared with water offering serving as control condition [6]. However, a pizza test meal cannot be considered as comparable to a standardized buffet test offering a balanced composition of a variety of widely differing foodstuff. Furthermore, the application of sucrose preloads of 200 and 300 kcal led to a higher total calorie consumption compared with water and sweet control [6]. As assumed, these amounts of glucose ingestion per se increased blood glucose concentrations to postprandial levels and are therefore not comparable to our study, in which fasting values of glucose and insulin were maintained. Accordingly, both low- and higher-calorie saccharide preloads trigger satiety. However, the high calorie content of the preloads applied in previous studies simply exceeded the energy saved during the meals.

Another important point, which may explain the difference between our results and previous findings, is the specific carbohydrate composition of the preload we used in our study. We applied a balanced composition of mono- and oligosaccharides, which ensure both a rapid and a sustained increase in blood glucose levels. Previous work applied sucrose [6–8], fructose [6], glucose [6], or fructose/glucose mixtures [7, 8], which all lead to a rapid and high rise in blood glucose concentrations but also a swift drop thereafter. In contrast, Yeomans et al. used maltodextrin [5]. The oligosaccharides contained therein result in a slightly delayed but more prolonged increase in blood glucose concentrations. However, a very low-calorie mixture of mono- and oligosaccharides has not been investigated as a preload, yet. This confirms our hypothesis that our specific amount and composition of mono- and oligosaccharides with its wide range of effects on blood glucose metabolism reduces food intake.

Moreover, the time interval between preload and test meal is also of decisive importance. Based on the ^31^P-MR spectroscopy results of our pilot study, we determined 30 min as the optimum time interval between capsule intake and test buffet. In contrast, the time interval between preload and test meal in the previous studies was 50 [8] and 60 [6, 7] minutes, respectively. Given the insight of our pilot study, which revealed that 30 min after preload application is the optimal time point for a meal, the persistence of the satiating effect of the preload one hour after its intake may be questionable, particularly since preloads in these studies were administered as mono- or disaccharides. In line, previous studies report a decrease in blood glucose levels 20–30 min after ingestion of the preload [7, 8], which confirms that this is the best time frame to affect food intake by a preload. Moreover, a meta-regression analysis revealed the intermeal interval as the predominant factor contributing to differences in energy compensation after a preload [9].

The exact underlying mechanisms of the observed effect of a 26 kcal saccharide preload on subsequent food intake cannot be explained by our human-experimental study. According to the glucostatic theory of appetite control [19], reduced glucose availability in hypothalamic satiety centers underlies the perception of hunger associated with the initiation of a meal, whereby increased glucose utilization in these brain regions leads to the perception of satiety followed by the termination of a meal. In fact, human-experimental studies show that transient and dynamic declines in blood glucose concentrations are associated with the initiation of meals and the onset of hunger [20–22]. When glucose levels rise, the release of anorexigenic peptides by glucose-excited neurons increases and the expression of orexigenic peptides by glucose-inhibited neurons decreases [23, 24]. Although it is not exactly known at which glucose concentrations these mechanisms are triggered, it seems conceivable that even a low-calorie preload-induced modest increase in blood glucose concentrations, which is still within the range of corresponding fasting values, leads to a rise in cerebral high-energy phosphates. In turn, this would result in the activation of hypothalamic satiety signals and accordingly reduce subsequent food intake [4]. The result of our study supports this assumption. Interestingly, the reduction in food intake was not only due to a lower carbohydrate consumption but also to diminished protein and fat intake in the saccharide compared with the placebo condition. In previous studies, the energy intake from the test meal was not differentially analyzed for macronutrients, whereby a comparison of our data is not possible in this respect. However, our result implies that glucose-induced satiety signals influence the intake of all macronutrients.

Finally, the results of the attention and concentration test (d2-R) should also be considered. We found improvements in the number of correct responses and the number of symbols processed at the second test run after capsule ingestion, which, however, did not differ between saccharide and placebo condition. The number of correct responses reflects concentration power, while the number of symbols processed is a parameter for the working speed [12]. Since there were no differences between both conditions, the improvement in concentration power and working speed is solely due to exercise gains through test repetition, as is often described in concentration tests [12]. In contrast, other studies have shown a positive effect of carbohydrate intake on attention [25] and cognitive performance [26]. However, the calorie content of the carbohydrate-containing beverage used in these studies was 145 kcal [25, 26], which is significantly higher than the calorie content of the saccharide preload in our study. Therefore, a positive effect of saccharide intake on attention and concentration is most likely only achieved through a larger amount of saccharides. In this context, the question arises whether the lack of effect on attention and concentration after saccharide administration argues against a central mechanism of cerebral energy levels in reducing food intake. However, since our attention and concentration test was only used as distraction from the actual study objective, we did not consider a high test sensitivity when choosing the d2-R for this purpose. We assume that food intake and cognitive function—although not compellingly linked to each other—are both related to cerebral energy levels but that our test sensitivity was not high enough to detect any differences in attention and concentration after an exiguous amount of the saccharide application.

In a clinical context, the observed effects of a low-calorie saccharide preload on food intake may lead to implications for a large number of people affected by metabolic diseases such as obesity or type 2 diabetes mellitus. The saccharide preload in our study had a very low energy content of only 26 kcal, which corresponds to about 7 g or one teaspoon of a saccharide mixture. A teaspoon of this mixture taken 30 min before a meal, therefore, has the potential to reduce energy intake. The administration in form of capsules in our study was intended to blind participants for taste, smell and appearance of the saccharide mixture vs. placebo. Of course, taking one teaspoon of this mixture would be more practical in a clinical use than taking this dose in 14 capsules. However, the long-term effects on potential weight loss in people with obesity need to be demonstrated by future studies. Assuming that the observed 9.3% reduction in calorie consumption forecasts the energy savings of an entire day, this would result in a significant overall reduction in food intake. Even if this effect only occurs with one meal per day, this calorie saving could have positive effects on body weight for individuals with obesity. However, this calculation is only a theory and a potential overall energy intake compensation could have occurred later in the day. Moreover, our saccharide preload may also have positive consequences on glucose metabolism in patients with type 2 diabetes since blood glucose and insulin levels did not raise above fasting values after preload application but still exert its appetite suppressing effect. Still, also this assumption needs to be verified in patients with diabetes.

However, there are some factors to consider, which may limit the interpretation of our results. We used a single-blind design for this basic research and we, therefore, cannot fully exclude any bias through the investigators as a double-blind study design could do. In addition, it would have been useful to systematically assess the efficacy of the blinding. Although the participants did not eat any food and drank only water after 8:00 PM the evening before experimental testing, it could also be beneficial for future studies to strictly control for physical activity, caffeine and alcohol consumption as well as food intake on the whole day before experimental testing. For future studies, it may also be useful to examine differences in meal duration between the two conditions that might influence eating speed and thus satiety perception. Furthermore, we only investigated the short-term effects of a saccharide preload on energy intake during a single meal and did not explore satiety and caloric intake throughout the remaining day. In addition, we only examined healthy male subjects. Whether these results are transferable to healthy females should be subject of further investigations. Moreover, it cannot be excluded that an impaired glucose metabolism, as found in obesity and diabetes, diminishes the observed effects on food intake or leads to a habituation effect. A possible adverse effect of the saccharide preload on postprandial hyperglycemia [27] is unlikely considering the small amount of saccharides administered in our study. Finally, information about absorption and degradation times of the saccharide preload would be beneficial for future applications.

Notwithstanding, our study shows that an exiguous saccharide preload of only 26 kcal suppresses subsequent food intake in men. This effect could be a promising approach for reducing caloric intake, avoiding the undesirable side effects of dieting, which should be investigated in ongoing research.

## Data Availability

The datasets generated and analyzed during the study are not publicly available but are available from the corresponding author on reasonable request.
